# TP53 status determines clinical significance of ERBB2 expression in ovarian cancer

**DOI:** 10.1038/sj.bjc.6602238

**Published:** 2004-11-16

**Authors:** J Kupryjańczyk, R Mądry, J Plisiecka-Hałasa, J Bar, E Kraszewska, I Ziółkowska, A Timorek, J Stelmachów, J Emerich, M Jędryka, A Płużańska, I Rzepka-Górska, K Urbański, J Zieliński, J Markowska

**Affiliations:** 1 Department of Molecular Pathology, The Maria Sklodowska-Curie Memorial Cancer Center, Institute of Oncology, ul. Roentgena 5, Warsaw 02-781, Poland; 2 Department of Pathology, Medical Academy and Brodnowski Hospital, ul. Kondratowicza 8, Warsaw 03-242, Poland; 3 Chair of Gynecologic Oncology, Medical Academy, ul. Lakowa 1/2, Poznan 61-878, Poland; 4 Department of Immunology, Medical Academy, ul. Dyrekcyjna 5/7, Wroclaw 50-528, Poland; 5 Department of Biostatistics, The Maria Sklodowska-Curie Memorial Cancer Center and Institute of Oncology, ul. Roentgena 5, Warsaw 02-781, Poland; 6 Department of Gynecologic Oncology, The Maria Sklodowska-Curie Memorial Cancer Center and Institute of Oncology, ul. Roentgena 5, Warsaw 02-781, Poland; 7 Department of Obstetrics and Gynecology, Medical Academy and Brodnowski Hospital, ul. Kondratowicza 8, Warsaw 03-242, Poland; 8 Department of Gynecologic Oncology, Medical Academy, ul. Kliniczna 1a, Gdansk 80-402, Poland; 9 Department of Gynecologic Oncology, Medical Academy, ul. Dyrekcyjna 5/7, Wroclaw 50-528, Poland; 10 Department of Gynecologic Oncology, Medical Academy, ul. Paderewskiego 4, Lodz 93-509, Poland; 11 Department of Gynecologic Oncology, Medical Academy, ul. Powstancow Wielkopolskich 72, Szczecin 70-111, Poland; 12 Department of Gynecologic Oncology, Institute of Oncology, ul. Garncarska 11, Krakow 31-115, Poland

**Keywords:** ERBB2, TP53, ovarian cancer

## Abstract

ERBB2 expression has been found in 19 to 44% of ovarian carcinomas; however, its predictive value has not been demonstrated, and trastuzumab has not found clinical application in ovarian cancer patients. We evaluated clinical significance of ERBB2 expression in relation to TP53 accumulation in ovarian carcinoma patients treated with platinum-based regimens. Immunohistochemical analysis with CB11 and a novel NCL-CBE356 antibody (against the internal and external domains of ERBB2, respectively) was performed on 233 tumours (FIGO stage IIB—IV); the US Food and Drug Administration-approved grading system with 0 to 3+ scale was used for evaluation, and the results were analysed by the Cox and logistic regression models. In all, 42% of the tumours expressed (category 1+, 2+ or 3+) either CB11 or CBE356 or both (CB11/CBE356 parameter). Associations between ERBB2 expression and clinical factors were observed only if tumours with staining category 1+ were grouped together with tumours showing staining categories 2+ and 3+. CB11/CBE356 parameter had a better predictive value than CB11 alone. CB11/CBE356 expression was negatively associated with platinum sensitivity (PS) in the TP53(−) group (*P*=0.022) and with disease-free survival (DFS) in the TP53(+) group (*P*=0.009). Our results may suggest that trastuzumab should be given postoperatively to patients with TP53(−)/ERBB2(+) ovarian carcinomas to enhance PS, and after completion of chemotherapy to patients with complete remission and TP53(+)/ERBB2(+) carcinomas to extend DFS time (in total to 30.4% of all patients analysed). Thus, novel criteria for ovarian cancer patient inclusion for clinical trials with trastuzumab should be considered and tested.

ERBB2 (HER-2) is a transmembrane tyrosine kinase receptor protein that belongs to the epidermal growth factor receptor family (HER-1, HER-2, HER-3, HER-4) (Slamon *et al*, 1989; Baselga and Albanell, 2001; Ross *et al*, 2003). The HER family participates primarily in transduction of proliferation signals from various ligands, and this process involves dimer formation between different HER receptors. The presence of HER-2 in a heterodimer results in more efficient stability and signalling (Baselga and Albanell, 2001; Ross *et al*, 2003). Overexpression of HER-2 results also in the formation of homodimers, which may be constitutively active (Baselga and Albanell, 2001).

The expression of HER-2 is an established prognostic factor in breast cancer. A monoclonal antibody against the external epitope of HER-2, that is, trastuzumab, is used for therapy of breast cancer patients whose tumours express high levels of HER-2, that is, 2+ and 3+ US Food and Drug Administration-approved category (Press *et al*, 2002; Ross *et al*, 2003).

Some data from cell lines overexpressing ERBB2 showed that therapy with trastuzumab and cisplatin had a synergistic effect (Hancock *et al*, 1991). This could create a possibility of more efficient therapy in ovarian cancer patients. However, despite relatively frequent ERBB2 overexpression or gene amplification in ovarian carcinomas (range 19–44%, Table 1

**Table 1:** Selected studies on clinical significance of ERBB2 expression in ovarian cancer patients (with positive results, the type of analysis is given, and a group in which the result has been found)

**References**	**No. of patients**	**Antibodies**	**Criterion of positivity Frequency of staining**	**CR or CR plus PR or PS**	**DFS**	**OS**
Berchuck *et al* (1990)	73	TA1	(3+) 32%	*P*<0.05 Fisher's test		*P*=0.001 univariate
Kacinski *et al* (1992)	72^a^	Anti-NEU	Weak, strong 3.1%		NS	NS
Rubin *et al* (1993)	105^a^	9G6	(3+), 24%			NS
Scambia *et al* (1993)	94^a^	Monoclonal pool	Weak, strong 35%		NS	NS
Meden *et al* (1994)	275		19%			YES multivariate
Fajac *et al* (1995)	52	Polyclonal	(>10%) 44%			NS
Felip *et al* (1995)	106^a^	CB11	(3+) 21.7%	*P*=0.004 χ^2^ test		*P*<0.001 (FIGO III, IV) multivariate
Van der Zee *et al* (1995)	89^a^	CB11	(>5%) 20%	NS	NS	NS
Tanner *et al* (1996)	79^a^	mRNA	Strong 20%			*P*=0.04 (FIGO III, IV) univariate
Meden *et al* (1998)	208^a^	Polyclonal, 9G6	>5% 22%			*P*=0.0003 univariate
Hengstler *et al* (1999)	77^a^	mRNA				*P*=0.036 multivariate
Ferrandina *et al* (2002)	76^a^	300G9	(3+) 21%	NS		NS
Present study	233^a^	CB11, 10A7	(1+, 2+, 3+) 42%	*P*=0.022 (TP53−) multivariate	*P*=0.009 (TP53+) multivariate	NS

DFS=disease-free survival; CR=complete remission; PR=partial remission; PS=platinum sensitivity; OS=overall survival; NS=not significant; no information on association means that it was not studied.

a Platinum-based chemotherapy.

), its clinical importance has been barely demonstrated (Berchuck *et al*, 1990; Kacinski *et al*, 1992; Rubin *et al*, 1993; Scambia *et al*, 1993; Meden *et al*, 1994; Fajac *et al*, 1995; Felip *et al*, 1995; Van der Zee *et al*, 1995; Tanner *et al*, 1996; Meden *et al*, 1998; Hengstler *et al*, 1999; Ferrandina *et al*, 2002). Only Berchuck *et al* (1990) and Felip *et al* (1995) observed lower frequency of complete remission (CR) in ERBB2-overexpressing tumours; however, they have not confirmed this by multivariate analysis. To date, trastuzumab has not found clinical application in ovarian cancer patients, neither combined with chemotherapy nor as monotherapy (Bookman *et al*, 2003).

We think that lack of clinical associations of ERBB2 overexpression in ovarian cancer studies may be due to (1) small group sizes (Table 1); (2) no regard to TP53 status and (3) overly restrictive criteria of ERBB2 overexpression.

We have recently shown that TP53 status may influence clinical importance of some molecular and clinical factors in ovarian carcinoma patients (Kupryjanczyk *et al*, 2003, 2004). The same may be true with regard to ERBB2. Some experimental studies have shown that wild-type TP53 protein limits or abrogates biological effects of ERBB2 stimulation. Both the growth inhibition and enhanced apoptosis were observed in wild-type TP53 cell lines after ERBB2 transfection. In the same conditions, TP53 mutant cells demonstrated enhanced growth (Casalini *et al*, 2001; Huang *et al*, 2002). Huang *et al* (2002) concluded that TP53 defects ‘played a permissive role in ERBB2 upregulation’, and ‘ERBB2 overexpression phenotype might in turn select for the survival of cells with p53 mutations’.

The common criteria of ERBB2 positivity (2+ and 3+) in tumour tissues were established with reference to ERBB2 expression in normal tissues. It has been shown that ERBB2 is normally expressed (1+) on epithelial cell membranes including ovarian epithelium and this is not due to gene amplification (Berchuck *et al*, 1990; Press *et al*, 1990; Rubin *et al*, 1993). On the other hand, Press *et al* (2002) evaluated CB11 expression (anti-ERBB2 antibody approved by FDA for clinical testing) (Press *et al*, 1994; Felip *et al*, 1995; Van der Zee *et al*, 1995; Bookman *et al*, 2003) in tumours with determined *ERBB2* gene amplification and mRNA expression, and found better CB11 sensitivity and accuracy for staining categories 1+ to 3+ than for 2+ and 3+ alone; the specificity was similar (98.6 *vs* 100%, respectively).

In the light of these findings, we aimed to evaluate the clinical significance of ERBB2 expression in a large group of advanced stage ovarian carcinomas, with the application of less stringent criteria of ERBB2 overexpression, and with respect to TP53 status. Another aim of the study was to test a novel CBE356 antibody (against external epitope of ERBB2) and to find out whether the evaluation of the internal (CB11) and external epitope of ERBB2 would be more clinically relevant than the evaluation of internal epitope only.

## MATERIALS AND METHODS

### Patients and tumours

The study was performed on archival material from 233 ovarian carcinoma patients operated on in the years 1987–1999. Medical records were critically reviewed by at least two clinicians. The material was carefully selected out of 548 cases submitted to meet the following criteria: no chemotherapy before staging laparotomy, adequate staging procedure, International Federation of Gynecologists and Obstetricians stage IIB to IV disease (Creasman, 1989), standard CP (cisplatin–cyclophosphamide or carboplatin–cyclophosphamide) or CAP chemotherapy (CP with the addition of doxorubicin), tumour tissue from the first laparotomy available, moderate (G2, 13%) or poor tumour differentiation (G3 and G4, 87%) and the availability of clinical data including residual tumour (RT) size and follow-up observation (Table 2

**Table 2:** Patient characteristics

	***N*=233**
*Age (years)*	
Range	24–77
Mean (s.d.)	53.2 (10.4)
	
*FIGO stage*	
IIB+IIC	17 (7%)
IIIA+IIIB	54 (23%)
IIIC	132 (57%)
IV	30 (13%)
	
*Residual tumour size*	
0	52 (22%)
>0⩽2 cm	60 (26%)
>2 cm	121 (52%)
	
*Chemotherapy*	
CP	167 (72%)
CAP	66 (28%)
	
*Response to chemotherapy*	
CR	123 (53%)
PR	36 (15%)
No change	8 (3%)
Progression	66 (28%)
	
Platinum sensitive	101 (43%)
Platinum resistant	132 (57%)
	
Recurrence rate in a CR group	98/123 (80%)
	
*Outcome*	
NED	28 (12%)
AWD	16 (7%)
DOD	184 (79%)
DOC	5 (2%)

CP=cyclophosphamide and cisplatin; CAP=CP plus doxorubicin; CR=complete remission; NED=no evidence of disease; AWD=alive with disease; DOD=died of disease; DOC=died of other causes.

).

All tumours were uniformly reviewed histopathologically, classified according to the criteria of the World Health Organisation (WHO) (Russell, 1994) and graded in a four-grade scale, according to the criteria given by Broders (Barber *et al*, 1975). There were 180 serous carcinomas (77%), 14 endometrioid (6%), 12 clear-cell type (5%), 14 undifferentiated (6%) and 13 other type carcinomas (6%).

Follow-up time ranged from 1.44 to 168.8 months (median 26 months), and 189 patients (81%) have died. Response to chemotherapy was determined retrospectively according to the WHO criteria (Miller *et al*, 1981). The evaluation was based on data from medical records describing the patient's clinical condition and CA125 levels in 3- to 4- week intervals. Complete remission was defined as disappearance of all clinical and biochemical symptoms of ovarian cancer evaluated after completion of first-line chemotherapy and confirmed at 4 weeks. Then, the patients were followed in 1- (33%), 2- (26%) or 3-month intervals (depending on the hospital centre) up to 6 or 12 months after completion of the first line of chemotherapy, and further in 2- to 3-month intervals up to 2 years after completion of chemotherapy. Within the CR group (*N*=123), we have identified a platinum-sensitive group (disease-free survival (DFS) longer than 6 months, 101 patients) (Christian and Trimble, 1994). The other tumours (partial remission – PR; progression – P; no change – NC), as well as the CR group with DFS shorter than 6 months, were described as resistant to cisplatin (Christian and Trimble, 1994) (Table 2) (Kupryjanczyk *et al*, 2003).

### Immunohistochemical analysis

Immunohistochemical procedure was performed on paraffin-embedded material after heat-induced epitope retrieval, according to the description given previously (Kupryjanczyk *et al*, 2003). We used CB11 (1 : 200) and the novel NCL-CBE356 (clone 10A7, 1 : 80, further called CBE356) monoclonal antibodies (both from Novocastra, Newcastle, UK) for the internal and external domains of the ERBB2 receptor, respectively. For detection of the TP53 protein, we used PAb1801 monoclonal antibody (1 : 500, Sigma-Genosys, Cambridge, UK) as described previously (Kupryjanczyk *et al*, 2003).

Briefly, deparaffinised sections were boiled 3 × 5 min (for ERBB2) or 2 × 5 min (for TP53) in a citrate buffer (pH 6.0) at 700 W in a microwave. Nonspecific tissue and endogenous peroxidase reactivity were blocked with 10% BSA and 3% H_2_O_2_, respectively. Tissue sections were incubated with primary antibodies overnight at 4°C (for ERBB2) or for 1 h (for TP53). Biotinylated goat anti-mouse IgG (1 : 1500, cat. no. 816), peroxidase-conjugated streptavidin (1 : 500, cat. no. 309) (both from Immunotech, Marseille, France) and DAB were used as a detection system. Cell lines, positive (SK-BR-3, MDA-175) and negative (MDA-231) for ERBB2 protein, provided in the Dako HercepTest™ (cat. no. K 5204, Dako, Glostrup, Denmark) served as controls for the procedure, as well as for the specificity of the CBE356 antibody. As a positive control for TP53, we used a tumour with a defined *TP53* gene missense mutation (Kupryjanczyk *et al*, 2000). Normal mouse IgGs of the same subclasses and concentrations as the primary antibodies served as negative controls, too.

### Evaluation of immunohistochemical stainings

The semiquantitative evaluation of immunohistochemical stainings was performed independently by two pathologists (JK, JB) without the knowledge of clinical data, and a consensus was reached in controversial cases. For the evaluation of ERBB2 expression, we applied the criteria approved by the US Food and Drug Administration, that is, lack of membranous staining or staining not exceeding 10% of cells was evaluated as negative; weak, barely perceptible incomplete membranous staining in at least 10% of cells was evaluated as 1+; weak or moderate staining in at least 10% of cells was evaluated as 2+; and strong staining in at least 10% of cells was evaluated as 3+ (Graziano, 1998; Jacobs *et al*, 1999). Five tumours representative of each ERBB2 staining category (in total 20) were stained once again for CBE356 expression 1 year after initial staining; the slides were evaluated without knowledge of initial results and the results were reproducible.

We combined CB11 expression with CBE356 expression (CB11/CBE356 parameter) by taking the highest result obtained with either antibody. For example, if a tumour was negative for CB11 and 3+ positive for CBE356, CB11/CBE356 parameter was scored 3+.

TP53 protein accumulation was described as present (more than 10% of positive cells) or absent (Kupryjanczyk *et al*, 2003).

### Statistical analysis

Associations between ERBB2 expression and clinicopathological variables were analysed by *χ*^2^ test. Probablity of survival and DFS were calculated using the Kaplan–Meier method (Kaplan and Meier, 1958). Overall and DFS time analyses were performed with multivariate Cox's proportional hazards models (Cox, 1972). Sensitivity to chemotherapy was evaluated with the multivariate logistic regression model. Significant parameters were selected using the backward selection technique, where factors not significant at 0.1 were drawn one by one out of the model. All tests were two-sided and the level of significance was set at 5%.

The statistical analysis included the following independent variables: age of patients, FIGO stage, RT size (0 *vs* ⩽2 cm, 0 *vs* >2 cm), histological type and histological grade. We evaluated clinical significance of CB11 expression, and that of CB11/CBE356 expression separately. The analysis was performed separately in the TP53(+) and TP53(−) subgroup, as well as in the whole group. The latter analysis was performed to check whether the lack of some ERBB2 associations in the TP53 subgroups was due to lower group sizes.

## RESULTS

### CB11 and CBE356 expressions and their associations

**Figure 1:**
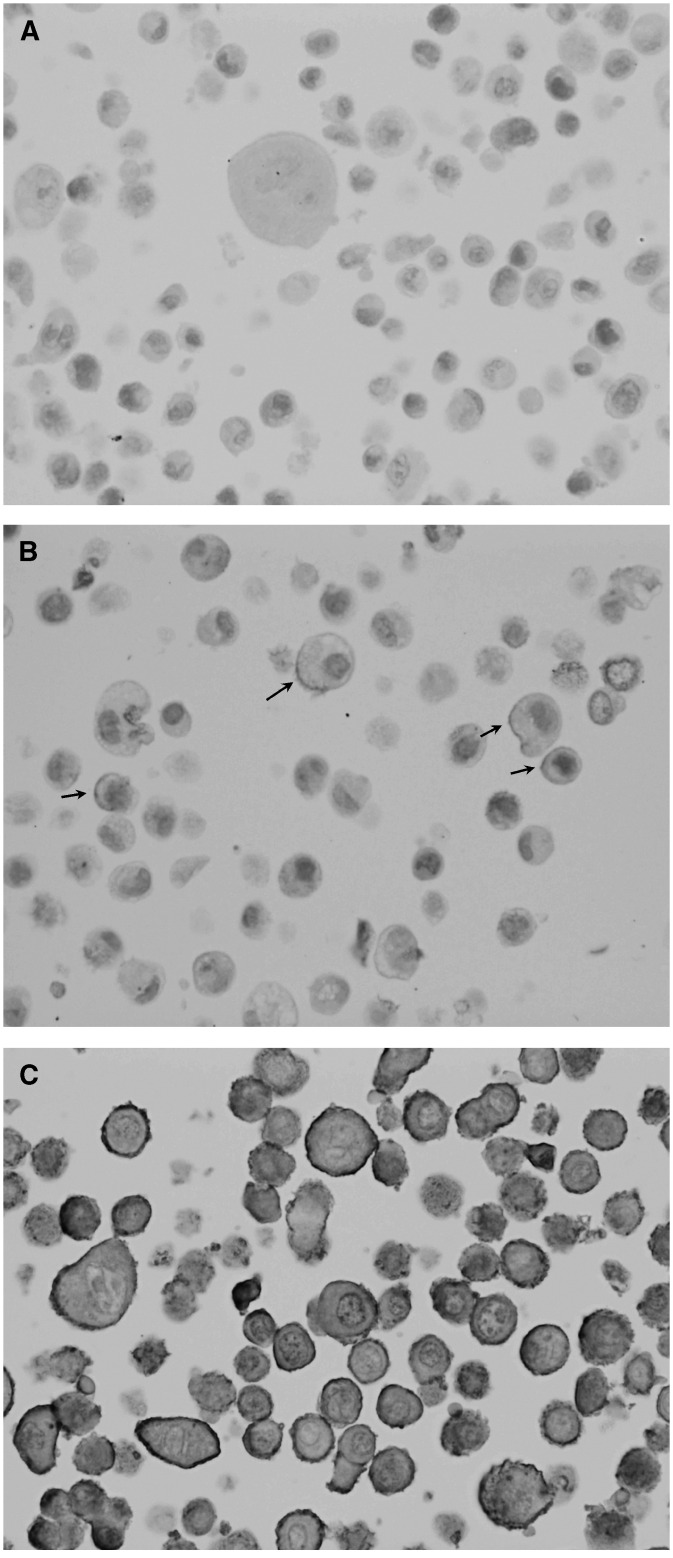
ERBB2 expression determined with NCL-CBE356 (10A7) monoclonal antibody in the control cell lines: (**A**) negative MDA-231 cell line, (**B**) 1+ positive MDA-175 cell line and (**C**) 3+ positive SK-BR-3 cell line.

Both antibodies gave membrane-bound (which is specific for ERBB2 receptor) and cytoplasmic staining and the latter was not taken into evaluation. Cytoplasmic staining for ERBB2 was usually observed also by other authors (Felip *et al*, 1995; Van der Zee *et al*, 1995; Bookman *et al*, 2003). SK-BR-3 cell line (3+) showed strong complete membranous staining in the majority of cells with both antibodies, and a variable cytoplasmic staining; MDA-175 cell line (1+) showed incomplete, barely perceptible membranous staining in a small percentage of the cells with both antibodies, as well as weak cytoplasmic staining in the Golgi region; MDA-231 cell line (negative for ERBB2) did not show specific staining with either antibody (Figure 1

).

CB11 and CBE356 expressions were highly associated with each other (*P*<0.00001); however, they did not completely overlap (Table 3

**Table 3:** Comparison of immunohistochemical CB11 expression with CBE356 (clone 10A7) expression in 233 ovarian carcinomas

	**No. of tumours**
*Both stainings positive*	35 (15%)
CB11 equal to 10A7	21
CB11 stronger than 10A7	8
CB11 weaker than 10A7	6
	
Both stainings negative	135 (58%)
	
*One staining negative*	63 (27%)
CB11 positive/ 10A7 negative	19
CB11 negative/10A7 positive	44

). CB11 expression was found in 54 tumours (23%, rates of tumours with category 1+, 2+ and 3+ were 7, 11 and 5%, respectively); CBE356 expression was found in 79 tumours (34%, a rate of tumours with category 1+, 2+ and 3+ was 13, 15.5 and 5.5%, respectively) (Figure 2

**Figure 2:**
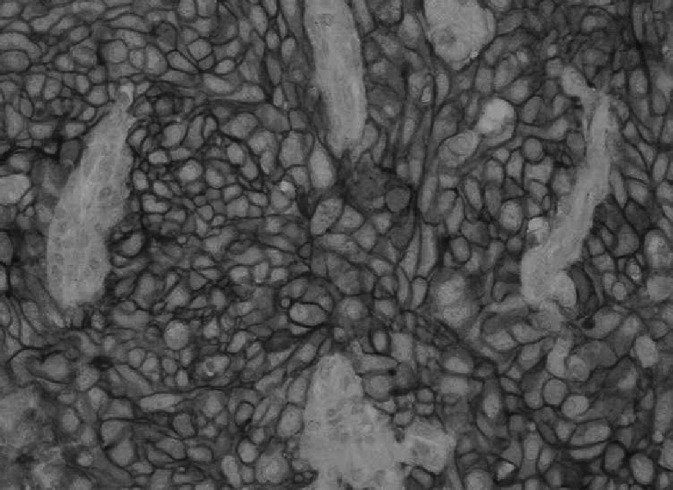
Strong membranous expression of ERBB2 in an ovarian carcinoma (NCL-CBE356 antibody, clone 10A7, streptavidin–biotin–peroxidase method, haematoxylin counterstain).

). In all, 42% of the carcinomas expressed either CB11 or CBE356 or both, and this is represented by CB11/CBE356 parameter; for this parameter, the rates of tumours with category 1+, 2+ and 3+ were 15, 20 and 7%, respectively. Thus, evaluation of both epitopes resulted in a much higher percentage of positive tumours.

To determine biological and clinical significance of category 1+ staining, first we compared tumours with categories 0, 1+ with those showing categories 2+, 3+, and then tumours with category 0 with those showing categories 1+ to 3+. Associations between ERBB2 expressions and biological or clinical factors were observed only if tumours with staining category 1+ were grouped together with tumours showing staining categories 2+ and 3+. Thus, the term ‘ERBB2 or CB11 or CB11/CBE356 expression’ will be further used in the meaning of staining categories 1+ to 3+.

CB11 expression was more frequent in the TP53(−) than in the TP53(+) carcinomas (30 *vs* 18%, *P*=0.04), as well as in FIGO IV tumours than in other clinical stages (43% expressors in stage IV, 19% in stage IIIC, 20% in IIIA and B, 29% in IIB and C, *P*=0.032). CB11/CBE356 expression did not show such associations. CB11/CBE356 expression was significantly less frequent in the serous (37%) than in other types of carcinoma (58.5%) (*P*=0.02). The rate of strong CB11/CBE356 expression (category 2+ and 3+) was particularly low in the serous type (23 *vs* 43.5%, *P*=0.01). CB11 and CB11/CBE356 expressions were neither associated with tumour differentiation nor with RT size.

### Associations of ERBB2 expression with clinical end points

CB11/CBE356 parameter (1+ to 3+) showed associations with DFS and platinum sensitivity (PS), while CB11 expression alone (1+ to 3+) was associated with PS at the border of significance only (Table 4

**Table 4:** Associations of ERBB2 expression with DFS (i.e. risk of recurrence, Cox's proportional hazards model) and probability of PS (logistic regression model) in the whole group of ovarian carcinomas, and in the TP53(+) and TP53(−) group

	**All patients, *N*=233, 189 deaths**		**TP53(−) group, *N*=97, 80 deaths**		**TP53(+) group, *N*=136, 109 deaths**	
		***P*-value**		****P*-value***		***P*-value**
*DFS*	CB11/CBE356^a^	0.012			CB11/CBE356	0.009
	RR=1.71				RR=2.15	
	95% CI (1.1, 2.6)				95% CI (1.2, 3.82)	
					CB11	0.07
	FIGO IIIA, B	0.036			*FIGO IIIA, B*	0.43
	FIGO IIIC	<0.001			FIGO IIIC	0.001
	FIGO IV	<0.001			FIGO IV	0.002
						
*PS*	CB11/CBE356	0.06	CB11/CBE356 OR=0.33 95% CI (0.13, 0.85)	0.022		
			CB11 OR=0.35 95% CI (0.12, 1.0)	0.05		
	RT⩽2 cm *vs* 0	0.014	RT⩽2 cm *vs* 0	0.03	RT⩽2 cm *vs* 0	0.31
	RT>2 cm *vs* 0	<0.001	RT>2 cm *vs* 0	0.001	RT>2 cm *vs* ⩽2 cm	<0.001

a CB11/CBE356 and CB11 categories 1+, 2+ and 3+ were compared with category 0. When CB11/CBE356 and CB11 categories 2+ and 3+ were compared with category 0 and 1+, no associations have been found. FIGO stages depicted were compared with FIGO IIB and C; RR=relative risk; OR=odds ratio; DFS=disease-free survival; PS=platinum sensitivity; CI=confidence interval. Detailed statistical results of RT and FIGO stage analysis have been given in previous publications (Kupryjanczyk *et al*, 2003, 2004).

). CB11/CBE356 expression enhanced 2.15 times the risk of recurrence in the TP53(+) group and not in the TP53(−) group (Figure 3

**Figure 3:**
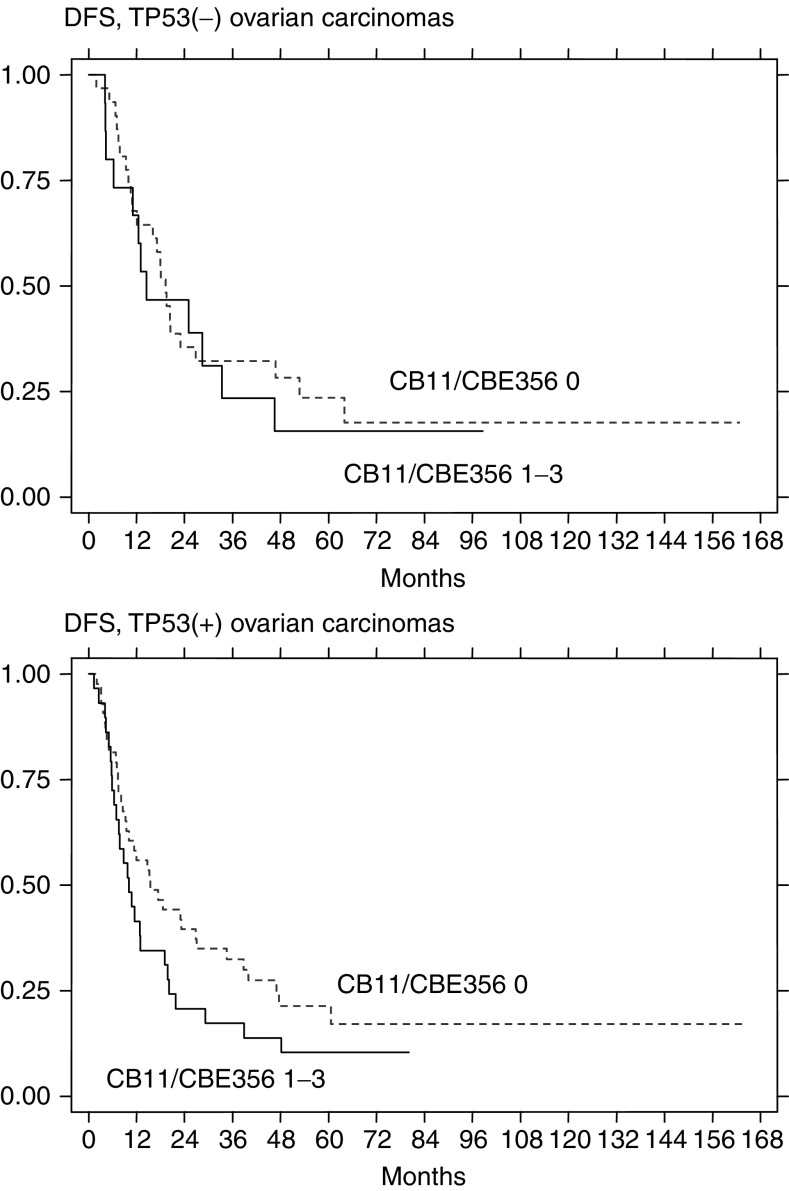
Kaplan–Meier curves for DFS in the TP53(−) and TP53(+) subgroups in relation to ERBB2 expression.

). In the whole tumour group, the association and the relative risk of recurrence were weaker than in the TP53(+) group (Table 4).

On the other hand, CB11/CBE356 expression diminished (0.33 times) the probability of PS in the TP53(−) group, and not in the larger TP53(+) group. In the whole tumour group, CB11/CBE356 expression showed only a borderline association with PS. CB11 expression alone showed up as a factor influencing PS in the TP53(−) group, while it was not associated with this end point in the whole group (Table 4).

CB11/CBE356 expression showed weaker associations with DFS or PS than clinical parameters. DFS was also associated with FIGO stage in the TP53(+) and in the whole group (lack of association in the TP53(−) group may be due to lower group size, Table 4). Residual tumour size (RT) was the only clinical parameter associated with PS in this analysis. Interestingly, complete debulking, when compared with RT ⩽2 cm, enhanced the probability of platinum-sensitive response in the TP53(−) group, while this has not been observed in the TP53(+) group (Table 4). Other factors evaluated did not show associations with DFS or PS. Overall survival and CR were not associated with ERBB2 expression. For full and detailed analysis of clinical factors in this patient group, the reader is referred to other publications (Kupryjanczyk *et al*, 2003, 2004).

## DISCUSSION

We have demonstrated the clinical significance of ERBB2 expression in ovarian carcinoma patients, that is, associations with PS and DFS, using a novel approach to the analysis that included: (1) evaluation in subgroups determined by TP53 status, (2) broader criteria for ERBB2 positivity and (3) a combined end-result from two monoclonal antibodies that identified the external and internal domains of ERBB2.

In our study, TP53 protein accumulation determined the clinical significance of ERBB2 expression. Platinum resistance related to ERBB2 expression was observed in the TP53(−) group only. Pegram *et al* (1997) reported that ERBB2 cDNA transfection into breast and ovarian cancer cell lines differentially influenced their sensitivity to cisplatin. We have confronted their findings with the known *TP53* status in some of those cell lines and the result is in accord with our observations. MCF-7/HER-2 and 2008/HER-2 cells that have wild-type TP53 showed resistance to cisplatin, while MDA-MB-435/HER-2, MDA-MB-231/HER-2 and Caov3-HER-2, which carry *TP53* gene mutation, did not differ in this respect from their progenitors without HER-2 expression; an exception is BT-20/HER-2 cell line that accumulates TP53 protein and is more resistant to cisplatin than parental cells without HER-2 expression (Barboule *et al*, 1995; Pegram *et al*, 1997). Mutated *TP53* most frequently results in profound alterations such as loss of cell-cycle control, impaired repair of DNA damage, apoptosis insufficiency as well as genomic instability. Possibly, ERBB2 overexpression in tumours with such molecular background is a minor additional alteration in terms of cell response to chemotherapy.

Furthermore, in our study, ERBB2 expression carried higher risk of recurrence (i.e. shorter DFS) in the TP53(+) group only and this may have an explanation in the findings of Casalini *et al* (2001) and Huang *et al* (2002). Tumour recurrence depends on cancer cell repopulation. Lincet *et al* (2000) reported that wild-type TP53 prevented cell repopulation by induction of P21^WAF1^. On the other hand, Huang *et al* (2002) observed that ERBB2 expression in wild-type TP53 MCF-7 cells induced TP53, P21^WAF1^, C-MYC and decreased BCL-2 expression (i.e. events promoting growth arrest and apoptosis), while this has not been observed after transfection of dominant-negative (i.e. mutant) TP53 (Casalini *et al*, 2001; Huang *et al*, 2002).

Our results suggest that the currently applied criteria for ERBB2 overexpression in ovarian cancer (i.e. 2+ and 3+) are too restrictive and from the clinical viewpoint it would be justified to include any ERBB2 membranous expression in above 10% of the tumour cells to positivity (or overexpression). One explanation of our findings might be that ERBB2 staining in paraffin-embedded material is artefactually diminished as compared with frozen tissue, and this is particularly true for weak and moderate expression (Slamon *et al*, 1989; Press *et al*, 1994). Our rate of 42% of the ovarian carcinomas expressing ERBB2 is close to 43–44% reported by Kacinski *et al* (1992) and Fajac *et al* (1995), who used similar criteria of positivity as in our study (Table 1).

A question related to this matter is whether tumours with weak ERBB2 expression would respond to anti-ERBB2 therapy. In both the clinical and the experimental studies on breast carcinomas, as well as on some ovarian cancer cell lines, tumour response to trastuzumab was proportional to the levels of ERBB2 expression (Xu *et al*, 1999; Vogel *et al*, 2002). While cell lines with low ERBB2 expression did not respond to an antibody therapy, fortunately, the antibody-mediated proliferation stimulation was not observed either (Xu *et al*, 1999). It is possible that the biological and clinical effect of anti-ERBB2 therapy in ovarian carcinoma cases with weak or higher ERBB2 expression will become apparent, if this is evaluated with respect to TP53 status.

Three immunohistochemical assays approved by FDA for clinical testing, that is, HercepTest assay, CB11 and 4D5 (‘Clinical Trials Assay’) and the Ventana Pathway CB11, as well as many other anti-ERBB2 antibodies (about 30), have different sensitivity and specificity when confronted with more objective methods of evaluation of gene amplification or expression (Press *et al* 1994, 2002; Jacobs *et al*, 1999; Roche and Ingle, 1999). We have chosen the known CB11 antibody against the internal domain, because it is approved for clinical testing (Press *et al*, 1994; Felip *et al*, 1995; Van der Zee *et al*, 1995; Bookman *et al*, 2003). We also wanted to test the novel 10A7 (NCL-CBE356) antibody against the external domain of ERBB2.

CB11 expression alone showed less clinical importance than the end-result from the two antibodies. During revision of this paper, for the purpose of discussion, we also checked whether CBE356/10A7 expression alone associates with PS or DFS; however, it did not show any clinical significance. Thus, it appears that concomitant evaluation of expression of the internal and external ERBB2 domains may be more clinically useful; possibly, similar results could be obtained with other anti-ERBB2 antibodies.

With the methods applied, we have possibly identified subgroups of ovarian cancer patients who may benefit from anti-ERBB2 therapy, and timing of such a therapy. Our results could suggest that trastuzumab should be given postoperatively to patients with TP53(−)/ERBB2(+) ovarian carcinomas (in our study 18%) to enhance PS; as shown above, postoperative trastuzumab might be useless in patients with TP53(+)/ERBB2(+) carcinomas because in the TP53(+) group, ERBB2 expression does not diminish PS. However, trastuzumab should be given to patients with TP53(+)/ERBB2(+) ovarian carcinomas who reached CR, that is, after completion of chemotherapy (in our study 12.4% of all patients), because in this group it might extend DFS time. According to our results (lack of associations with overall survival), the group of patients with TP53(+)/ERBB2(+) tumours who did not reach CR would not qualify for trastuzumab treatment. Thus, novel criteria for ovarian cancer patient inclusion for clinical trials with trastuzumab should be considered and further tested. If confirmed, our results could create a perspective of the additional treatment for 30% of patients with advanced stage ovarian carcinomas.

## References

[bib1] Barber HR, Sommers SC, Snyder R, Kwon TH (1975) Histologic and nuclear grading and stromal reactions as indices for prognosis in ovarian cancer. Am J Obstet Gynecol 121: 795–807 1092171

[bib2] Barboule N, Mazars P, Baldin V, Vidal S, Jozan S, Martel P, Valette A (1995) Expression of p21WAF1/CIP1 is heterogeneous and unrelated to proliferation index in human ovarian carcinoma. Int J Cancer 63: 611–615 7591274 10.1002/ijc.2910630502

[bib3] Baselga J, Albanell J (2001) Mechanism of action of anti-HER2 monoclonal antibodies. Ann Oncol 12(Suppl 1): S35–S41 11521720 10.1093/annonc/12.suppl_1.s35

[bib4] Berchuck A, Kamel A, Whitaker R, Kerns B, Olt G, Kinney R, Soper JT, Dodge R, Clarke-Pearson DL, Marks P (1990) Overexpression of HER-2/*neu* is associated with poor survival in advanced epithelial ovarian cancer. Cancer Res 50: 4087–4091 1972347

[bib5] Bookman MA, Darcy KM, Clarke-Pesrson D, Boothby RA, Horowitz IR (2003) Evaluation of monoclonal humanized anti-HER2 antibody, transtuzumab, in patients with recurrent or refractory ovarian or primary peritoneal carcinoma with overexpression of HER2: a phase II trial of the gynecologic oncology group. J Clin Oncol 21: 283–290 12525520 10.1200/JCO.2003.10.104

[bib6] Casalini P, Botta L, Ménard S (2001) Role of p53 in HER2-induced proliferation or apoptosis. J Biol Chem 276: 12449–12453 11278558 10.1074/jbc.M009732200

[bib7] Christian MC, Trimble EL (1994) Salvage chemotherapy for epithelial ovarian carcinoma. Gynecol Oncol 55(Suppl): S143–S150 7835799 10.1006/gyno.1994.1354

[bib8] Cox DR (1972) Regression models and life tables. J Roy Stat Soc 34: 187–220

[bib9] Creasman WJ (1989) Announcement, FIGO stages 1988, revisions. Gynecol Oncol 35: 125–127

[bib10] Fajac A, Benard J, Lhomme C, Rey A, Duvillard P, Rochard F, Bernaudin JF, Riou G (1995) *c-erbB2* gene amplification and protein expression in ovarian epithelial tumours: evaluation of their respective prognostic significance by multivariate analysis. Int J Cancer 64: 146–151 7615357 10.1002/ijc.2910640213

[bib11] Felip E, Del Campo JM, Rubio D, Vidal MT, Colomer R, Bermejo B (1995) Overexpression of c-erbB-2 in epithelial ovarian cancer. Prognostic value and relationship with response to chemotherapy. Cancer 75: 2147–2152 7697606 10.1002/1097-0142(19950415)75:8<2147::aid-cncr2820750818>3.0.co;2-8

[bib12] Ferrandina G, Ranelletti OF, Lauriola L, Fanfani F, Legge F, Mottolese M, Nicotra MR, Natali PG, Zakut VH, Scambia G (2002) Cyclooxygenase-2 (COX-2), epidermal growth factor receptor (EGFR), and Her-2/neu expression in ovarian cancer. Gynecol Oncol 85: 305–310 11972392 10.1006/gyno.2002.6620

[bib13] Graziano C (1998) HER-2 breast assay, linked to Herceptin, wins FDA's okay. CAP Today 12: 13–16 10187049

[bib14] Hancock MC, Langton BC, Chan T, Toy P, Monahan JJ, Mischak RP, Shawver LK (1991) A monoclonal antibody against the c-erbB-2 protein enhances the cytotoxicity of cisdiammi-nedichloroplatinum against human breast and ovarian tumor cell lines. Cancer Res 51: 4575–4580 1678683

[bib15] Hengstler JG, Lange J, Kett A, Dornhofer N, Meinert R, Arand M, Knapstein PG, Becker R, Oesch F, Tanner B (1999) Contribution of c-erbB-2 and topoisomerase II*α* to chemoresistance in ovarian cancer. Cancer Res 59: 3206–3214 10397267

[bib16] Huang GC, Hobbs S, Walton M, Epstein RJ (2002) Dominant negative knockout of p53 abolishes ErbB2-dependent apoptosis and permits growth acceleration in human breast cancer cells. Br J Cancer 86: 1104–1109 11953857 10.1038/sj.bjc.6600219 PMC2364174

[bib17] Jacobs TW, Gown AM, Yaziji H, Barnes MJ, Schnitt SJ (1999) Specificity of Hercep Test in determining HER-2/*neu* status of breast cancers using the United States Food and Drug Administration-approved scoring system. J Clin Oncol 17: 1983–1987 10561248 10.1200/JCO.1999.17.7.1983

[bib18] Kacinski BM, Mayer AG, King BL, Carter D, Chambers SK (1992) *NEU* protein overexpression in benign, borderline, and malignant ovarian neoplasms. Gynecol Oncol 44: 245–253 1347282 10.1016/0090-8258(92)90051-j

[bib19] Kaplan EL, Meier P (1958) Non-parametric estimation from incomplete observations. J Am Stat Assoc 53: 457–481

[bib20] Kupryjanczyk J (2004) TP53 status determines prognostic and predictive factors in ovarian carcinomas. In Trends in Ovarian Cancer Research Bardos AP (ed) New York: Nova Science Publishers Inc., (in press)

[bib21] Kupryjanczyk J, Dansonka-Mieszkowska A, Szymanska T, Karpinska G, Rembiszewska A, Rusin M, Konopinski R, Kraszewska E, Timorek A, Yandell DW, Stelmachow J (2000) Spontaneous apoptosis in ovarian carcinomas: a positive association with *p53* gene mutation is dependent on growth fraction. Br J Cancer 82: 579–583 10682669 10.1054/bjoc.1999.0967 PMC2363315

[bib22] Kupryjanczyk J, Szymanska T, Mądry R, Timorek A, Stelmachow J, Karpinska G, Rembiszewska A, Ziolkowska I, Kraszewska E, Debniak J, Emerich J, Ulanska M, Pluzanska A, Jedryka M, Goluda M, Chudecka-Glaz A, Rzepka-Gorska I, Klimek M, Urbanski K, Breborowicz J, Zielinski J, Markowska J (2003) Evaluation of clinical significance of TP53, BCL-2, BAX and MEKI expression in 229 ovarian carcinomas treated with platinum-based regimen. Br J Cancer 88: 848–858 12644821 10.1038/sj.bjc.6600789 PMC2377076

[bib23] Lincet H, Poulain L, Remy JS, Deslandes E, Duigou F, Gauduchon P, Staedel C (2000) The p21(cip1/waf1) cyclin-dependent kinase inhibitor enhances the cytotoxic effect of cisplatin in human ovarian carcinoma cells. Cancer Lett 161: 17–26 11078909 10.1016/s0304-3835(00)00586-3

[bib24] Meden H, Marx D, Rath W (1994) Overexpression of the oncogene c-erb B2 in primary ovarian cancer: evaluation of the prognostic value in a Cox proportional hazards multiple regression. Int J Gynecol Pathol 13: 45–53 7906681 10.1097/00004347-199401000-00006

[bib25] Meden H, Marx D, Roegglen T, Schauer A, Kuhn W (1998) Overexpression of the oncogene c-erbB-2 (HER2/neu) and response to chemotherapy in patients with ovarian cancer. Int J Gynecol Pathol 17: 61–65 9475194 10.1097/00004347-199801000-00011

[bib26] Miller AB, Hogestraeten B, Staquet M, Winkler A (1981) Reporting results of cancer treatment. Cancer 47: 207–214 7459811 10.1002/1097-0142(19810101)47:1<207::aid-cncr2820470134>3.0.co;2-6

[bib27] Pegram MD, Finn RS, Arzoo K, Beryt M, Pietras RJ, Slamon DJ (1997) The effect of HER-2/*neu* overexpression on chemotherapeutic drug sensitivity in human breast and ovarian cancer cells. Oncogene 15: 537–547 9247307 10.1038/sj.onc.1201222

[bib28] Press MF, Cordon-Cardo C, Slamon DJ (1990) Expression of the HER-2/*neu* proto-oncogene in normal human adult and fetal tissues. Oncogene 5: 953–962 1973830

[bib29] Press MF, Hung G, Godolphin W, Slamon DJ (1994) Sensitivity of HER-2/*neu* antibodies in archival tissue samples: potential source of error in immunohistochemical studies of oncogene expression. Cancer Res 54: 2771–2777 7909495

[bib30] Press MF, Slamon DJ, Flom KJ, Park J, Zhou JY, Bernstein L (2002) Evaluation of HER-2/*neu* gene amplification and overexpression: comparison of frequently used assay methods in a molecularly characterized cohort of breast cancer specimens. J Clin Oncol 20: 3095–3105 12118023 10.1200/JCO.2002.09.094

[bib31] Roche PC, Ingle JN (1999) Increased HER2 with US Food and Drug Administration-approved antibody. J Clin Oncol 17: 434 (letter) 10.1200/JCO.1999.17.1.434 10458264

[bib32] Ross JS, Fletcher JA, Linette GP, Stec J, Clark E, Ayers M, Symmans WF, Pusztai L, Bloom KJ (2003) The HER-2/*neu* gene and protein in breast cancer 2003: biomarker and target of therapy. Oncologist 8: 307–325 12897328 10.1634/theoncologist.8-4-307

[bib33] Rubin SC, Findstad CL, Wong GY, Almadrones L, Plante M, Lloyd KO (1993) Prognostic significance of HER-2/*neu* expression in advanced epithelial ovarian cancer: A multivariate analysis. Am J Obstet Gynecol 168: 162–169 8093588 10.1016/s0002-9378(12)90907-2

[bib34] Russell P (1994) Surface epithelial–stromal tumors of the ovary. In Blaustein's Pathology of the Female Genital Tract Kurman RJ (ed) pp 705–782. Berlin, Heidelberg, New York: Springer-Verlag

[bib35] Scambia G, Panici PB, Ferrandina G, Battaglia F, Baiocchi G, Di Stefano P, Tinari N, Coronetta F, Piantelli M, Natali P, Iacobelli S, Mancuso S (1993) Expression of HER-2/neu oncoprotein, DNA-ploidy and S-phase fraction in advanced ovarian cancer. Int J Gynaecol Cancer 3: 271–278 10.1046/j.1525-1438.1993.03050271.x 11578357

[bib36] Slamon DJ, Godolphin W, Jones LA, Holt JA, Wong SG, Keith DE, Levin WJ, Stuart SG, Udove J, Ullrich A, Press MF (1989) Studies of the HER-2/*neu* proto-oncogene in human breast and ovarian cancer. Science 244: 707–712 2470152 10.1126/science.2470152

[bib37] Tanner B, Kreutz E, Weikel W, Meinert R, Oesch F, Knapstein PG, Becker R (1996) Prognostic significance of c-erbB-2 mRNA in ovarian carcinoma. Gynecol Oncol 62: 268–277 8751560 10.1006/gyno.1996.0226

[bib38] Van der Zee AG, Hollema H, Suurmeijer AJ, Krans M, Sluiter WJ, Willemse PH, Aalders JG, de Vries EG (1995) Value of P-glycoprotein, glutathione *S*-transferase pi, c-erbB-2, and p53 as prognostic factors in ovarian carcinomas. J Clin Oncol 13: 70–78 7799045 10.1200/JCO.1995.13.1.70

[bib39] Vogel CL, Cobleigh MA, Tripathy D, Gutheil JC, Harris LN, Fehrenbacher L, Slamon DJ, Murphy M, Novotny WF, Burchmore M, Shak S, Stewart SJ, Press M (2002) Efficacy and safety of trastuzumab as a single agent in first-line treatment of HER2-overexpressing metastatic breast cancer. J Clin Oncol 20: 719–726 11821453 10.1200/JCO.2002.20.3.719

[bib40] Xu F, Yu Y, Le XF, Boye C, Mills GB, Bast Jr RC (1999) The outcome of heregulin-induced activation of ovarian cancer cells depends on the relative levels of HER-2 and HER-3 expression. Clin Cancer Res 5: 3653–3660 10589783

